# ICOS-Expressing Lymphocytes Promote Resolution of CD8-Mediated Lung Injury in a Mouse Model of Lung Rejection

**DOI:** 10.1371/journal.pone.0072955

**Published:** 2013-08-13

**Authors:** Qiang Wu, Gail J. Gardiner, Elizabeth Berry, Sarah R. Wagner, Tiffany Lu, Bryan S. Clay, Tamson V. Moore, Caroline M. Ferreira, Jesse W. Williams, Andrew D. Luster, Benjamin D. Medoff, Judy L. Cannon, Anne I. Sperling, Rebecca A. Shilling

**Affiliations:** 1 Center for Immunobiology, Division of Pulmonary and Critical Care Medicine, Department of Medicine and Department of Microbiology and Immunology, Indiana University School of Medicine, Indianapolis, Indiana, United States of America; 2 Committee on Immunology & Section of Pulmonary and Critical Care Medicine, Department of Medicine, the University of Chicago, Chicago, Illinois, United States of America; 3 Center for Immunology and Inflammatory Diseases, Division of Rheumatology, Allergy and Immunology, Massachusetts General Hospital and Harvard Medical School, Charlestown, Massachusetts, United States of America; 4 Pulmonary and Critical Care Unit, Massachusetts General Hospital and Harvard Medical School, Boston, Massachusetts, United States of America; Université Paris Descartes, France

## Abstract

Acute rejection, a common complication of lung transplantation, may promote obliterative bronchiolitis leading to graft failure in lung transplant recipients. During acute rejection episodes, CD8^+^ T cells can contribute to lung epithelial injury but the mechanisms promoting and controlling CD8-mediated injury in the lung are not well understood. To study the mechanisms regulating CD8^+^ T cell–mediated lung rejection, we used a transgenic model in which adoptively transferred ovalbumin (OVA)-specific cytotoxic T lymphocytes (CTL) induce lung injury in mice expressing an ovalbumin transgene in the small airway epithelium of the lungs (CC10-OVA mice). The lung pathology is similar to findings in humans with acute lung transplant. In the presence of an intact immune response the inflammation resolves by day 30. Using CC10-OVA.RAG^-/-^ mice, we found that CD4^+^ T cells and ICOS^+/+^ T cells were required for protection against lethal lung injury, while neutrophil depletion was not protective. In addition, CD4^+^Foxp3 ^+^ ICOS^+^ T cells were enriched in the lungs of animals surviving lung injury and ICOS^+/+^ Tregs promoted survival in animals that received ICOS^-/-^ T cells. Direct comparison of ICOS^-/-^ Tregs to ICOS^+/+^ Tregs found defects in vitro but no differences in the ability of ICOS^-/-^ Tregs to protect from lethal lung injury. These data suggest that ICOS affects Treg development but is not necessarily required for Treg effector function.

## Introduction

CD8^+^ T cells are important mediators of the adaptive immune response to pathogens and tumors but their function as cytotoxic T lymphocytes (CTL) can also lead to immunopathology. During viral infections bystander activation and recruitment of inflammatory cells can lead to prolonged lung injury and collateral damage to the tissue [1]. For lung transplants, CD8^+^ T cells may play a critical role in driving the alloimmune response that leads to acute rejection and may promote chronic rejection [2–6]. Increased pro-inflammatory cytokine producing CD8^+^ T cells can be found in the bronchoalveolar lavage (BAL) during episodes of acute lung transplant rejection in humans [7]. CD8^+^ T cells have also been found to be capable of inducing rejection in the lung independent of CD4^+^ T cells in a mouse model of orthotopic lung transplant [7,8]. However, the mechanisms to control CD8^+^ mediated injury and damage to lung tissue are not well understood.

Several types of T cell subpopulations have been shown to display immunoregulatory capacity [9]. Natural T regulatory cells (Tregs) represent approximately 5-10% of CD4^+^ T cells and express the intracellular transcription factor Foxp3 [10,11]. Accumulating evidence from both animal models and clinical studies demonstrate that Tregs are important in both the induction and maintenance of allograft tolerance [12–14]. The localization of Tregs in the graft after transplant is important for effectively controlling aggressive immune reactivity to the graft [15–18]. Investigators have found that stable lung transplant recipients have an increased percentage of Tregs in bronchoalveolar lavage (BAL) fluid compared to subjects with subsequent lung allograft dysfunction, suggesting Tregs may control the alloimmune response in the lung [19]. A better understanding of the mechanisms controlling Treg function and expansion may lead to better therapies for lung transplant recipients.

Costimulatory molecules are known to regulate the development and function of Tregs [20]. Mouse and human Tregs express the negative regulator CTLA-4 and blockade of CTLA-4 leads to a decrease in alloantigen-specific Treg-mediated suppression [21–25]. CD28 is also known to be important for Treg differentiation and homeostasis as targeted mutations in CD28, as well as blockade of the CD28/B7-1/B7-2 pathway during development result in a remarkable decrease in Treg numbers [26,27]. More recently, inducible costimulator (ICOS) has been found to be required for optimal Treg function and development [28–31]. ICOS has been found to be expressed by Tregs infiltrating the lung during viral infection and is suggested to play a role in controlling CD8-mediated inflammation in the skin [30,31]. However, the requirement for ICOS-expression by Tregs in the lung to control CD8-mediated lung injury is not known.

In this study, we have used a previously developed model of antigen-specific T cell mediated acute bronchiolitis using transgenic animals expressing transmembrane ovalbumin (OVA) in the small airway epithelium of the lung under the control of the Clara cell promoter (CC10) [32]. Adoptive transfer of in vitro activated OVA-specific CD8^+^ T cells from OVA TCR transgenic (OT-I) mice induces lung pathology similar to findings in humans with acute lung transplant rejection and virus- induced lung injury. By adjusting the conditions for activated OT-I transfer, we have used this model in CC10-OVA.RAG^-/-^ mice to dissect the mechanism by which bystander cells modulate lung rejection. We found that neutrophils were not required for lethal lung injury and ICOS^+^ Tregs were significantly increased in the lung during acute inflammation. Moreover, ICOS^-/-^ lymphocytes were not sufficient to prevent death in CC10-OVA.RAG^-/-^ mice but wild-type Tregs could rescue this defect. These data suggested that ICOS expression regulated the ability of CD4^+^ Tregs to suppress CD8 T cell mediated lung injury.

However, when equal numbers of ICOS^-/-^ Tregs were transferred compared to wild-type Tregs, ICOS^-/-^ Tregs were able to prevent lethal lung injury. Our data suggest ICOS may be required for Treg development but is not necessarily required for Treg effector function in the lung.

## Materials and Methods

### Ethics Statement

All animal studies were done in concordance with principles set forth by the Animal Welfare Act and the National Institutes of Health guidelines for the care and use of animals in biomedical research. All experimental mouse protocols were approved by the Institutional Animal Care and Use Committees of the University of Chicago and Indiana University School of Medicine.

### Animals

C57BL/6 mice (4- to 6-wk-old) were purchased from the National Cancer Institute (Frederick, MD), Harlan Laboratories (Indianapolis, IN) or bred in house. B6.CC10-OVA (Thy1.2, C57BL/6) mice were generated as previously described [32]. OT-I. Thy1.1 T cell receptor transgenic mice were a gift of Dr. Yang-Xin Fu (University of Chicago). CC10-OVA mice were crossed to B6.RAG-1^-/-^ mice purchased from Jackson Labs (Bar Harbor, ME to generate B6.CC10-OVA.RAG^-/-^mice. ICOS^-/-^ mice), were backcrossed to C57Bl/6 for greater than 10 generations or purchased from Jackson Labs (Bar Harbor, ME). All mice were housed under specific pathogen-free conditions in the animal care facility at University of Chicago or Indiana University School of Medicine.

### Activated OT-I T cell preparation

Preparation of OT-I CD8^+^ CTL was modified from previously published methods [32,33]. Cellular suspensions were prepared from the spleen and lymph nodes of OT-I. Thy1.1 mice and cultured for 3 days with 1µg/ml OVA peptide antigen (SIINFEKL), 1 µg/ml anti-CD28 (BioXcell), 25U/ml murine IL-2 (NIH), and 10 ng/ml recombinant mouse IL-12 (Invitrogen) in 10% FCS DMEM medium in 24-well plates. Cells were then transferred to 75 ml flask and cultured with either 12.5U/ml IL-2 (low IL-2) or 25U/ml (high IL-2). OT-I CTL for transfer were harvested 24 hours later.

### Adoptive transfer and depletion experiments

Activated OT-I. Thy1.1 CTL were prepared as mentioned above. Live cells were separated by centrifugation through a ficoll gradient. Cells were washed and resuspended in PBS, and the indicated numbers of cells were injected i.v. via the retro-orbital venous plexus. Unfractionated lymphocytes transfer: spleen and lymph nodes were harvested from C57Bl/6 or ICOS^-/-^ mice to generate single cell suspensions. Cells were washed, red cells lysed with ACK lysis buffer (spleen), centrifuged through ficoll gradient to harvest live cells and washed and resuspended in PBS. 50x10^6^ single cells were transferred via retro-orbital sinus 2 days prior to the intravenous administration of activated OT-I T cells. CD4^+^ T cell depleted lymphocytes: C57Bl/6 spleen and lymph nodes were harvested and processed into single-cell suspensions. Cells were incubated with anti-CD4 antibody (RL172.4) at 4^o^C for 1 hour followed by incubation with rabbit complement for 30 minutes at 37^o^C and recovery of non-CD4^+^ T cells by ficoll. Depletion was confirmed by flow cytometry and over 90% of CD4^+^ T cells were depleted. The CD4-depleted cells were resuspended in PBS and 50x10^6^ single cells were transferred i.v. 2 days prior to the intravenous injection of OT-I T cells. Alternatively, CD4^+^ T cells were depleted using CD4 depletion kit according to manufacturer’s instructions (Miltenyi Biotec). Tregs were isolated from spleen and lymph node using the CD4^+^CD25^+^ Regulatory T Cell Isolation Kit according to manufacturer’s instructions (Miltenyi Biotec). Tregs were transferred i.v. along with ICOS^-/-^ splenocytes or CD4-depleted splenocytes two days prior to the transfer of OT-I T cells. Neutrophil depletion: mice were injected i.p. 2 days before OT-I transfer and 3 and 5 days after transfer with 300 µg anti-Ly6G antibodies (1A8, BioXcell) or 300 µg isotype control antibody (2A3, BioXcell). Mice were followed daily for morbidity and were counted as a mortality if >20% weight loss was found.

### Lung histology, digestions and bronchoalveolar lavage (BAL)

Lungs were fixed in 10% formalin and embedded in paraffin for H&E staining. Lung cell preparation has been previously described [34]. Lung fragments were digested with 150u/ml collagenase I for 30 min at 37° C and then gently pipetted for 1 min. Single-cell suspensions were stained for flow cytometry. For BAL, lungs were lavaged with four consecutive washes with 0.8 ml PBS, and 3 ml of pooled lavage fluid was recovered. White cells were counted and stained with targeted antibodies for flow cytometry.

### Flow cytometry

Cell surface staining: cells from lung and BAL were incubated with Fc block (2.4G2 antibody) and then stained with APC*-*anti-CD3, APC-Cy7-anti-CD8, PE-anti-Gr1 (Ebioscience), and PE-Cy7-anti-Thy1.1 (Biolegend) in FACS buffer (PBS containing 2% BSA and 0.01% sodium azide) on ice for 30 min. Neutrophils were identified by characteristic size by FSC and Gr1^Hi^ staining. Intracellular staining for Foxp3: staining was conducted using the anti-Mouse/Rat Foxp3 Staining Set APC according to the manufacturer’s instructions, with some modifications (eBioscience). Lung cells were washed after incubation with FITC-anti-ICOS (from Biolegend), PerCp-Cy5.5-anti-CD4, PE-anti-CD25, and resuspended in FoxP3 fixation/permeabilization buffer (eBioscience), and then incubated for 10 min at 4° C, washed twice in FoxP3 permeabilization buffer (eBioscience), and stained with anti-Foxp3-APC in Foxp3 permeabilization buffer for 1 h at 4° C. Cells were then washed twice in Foxp3 permeabilization buffer and resuspended in FACS buffer. Intracellular cytokine staining: cells were incubated with PMA (20ng/ml) and ionomycin (2 µg/ml) for 5 Hours and Brefeldin A (10µg/ml) was added for the last 4 hours of culture. Cells were stained with surface stains for CD8, Thy1.1 and CD3, fixed with 2% PFA and then permeabilized with 0.1% saponin buffer and stained intracellularly with anti-Interferon- γ, anti-TNF-α or the respective isotype controls. Cells were acquired with an LSR-II flow cytometer (BD Biosciences) and analyzed with FlowJo software (Tree Star, Ashland, OR).

### Treg in vitro cytokine expression: ELISA and qRT-PCR

Spleen and lymph node were harvested from C57BL/6 ICOS^+/+^ and ICOS^-/-^ mice, and Treg were separated using mouse CD4^+^CD25^+^ Regulatory T Cell Isolation Kit (Miltenyi Biotec). 4 x 10^5^ cells were stimulated with 0.5µg/mL anti-CD3 and 100 U IL-2 and cultured at 37° C for 72 hours [35]. Culture supernatants were harvested and analyzed with Mouse IL-10 ELISA MAX Standard Sets (BioLegend). For qRT-PCR, RNA was isolated from ex vivo and activated CD4^+^CD25^+^ Treg using RNeasy Mini Kit (Qiagen) and stored at -80° C. cDNA was generated with qScript cDNA SuperMix, and qRT-PCR was immediately performed using PerfeCTa^TM^ SYBR Green FastMix^TM^, Low ROX^TM^ (Quanta Biosciences) and the following primers: (Il10) 5’- GGTTGCCAAGCCTTATCGGA-3’, 5’-ACCTGCTCCACTGCCTTGCT-3’; (Tgfβ1) 5’-TGACGTCACTGGAGTTGTACGG-3’, 5’-GGTTCATGTCATGGATGGTGC-3’; (Ebi3) 5’-AGCAGCAGCCTCCTAGCCT-3’, 5’-ACGCCTTCCGGAGGGTC-3’; (Il12a) 5’- TGGCTACTAGAGAGACTTCTTCCACAA-3’, 5’-GCACAGGGTCATCATCAAAGAC-3’; and (β-actin) 5’- GGCTGTATTCCCCTCCATCG-3’, 5’-CCAGTTGGTAACAATGCCATGT-3’. Relative quantification (RQ) values were calculated relative to β-actin and presented as fold change compared to ICOS^+/+^ unstimulated samples.

### Treg in vitro proliferation and survival analysis

Spleen and lymph node were harvested from C57BL/6 ICOS^+/+^ and ICOS^-/-^ mice, and single-cell suspensions were prepared. Cells were resuspended and labeled with 2 µM CFSE (Sigma) for 10 minutes at room temperature. Labeled cells were left unstimulated or stimulated with 0.01 µg/ml or 0.1 µg/ml anti-CD3ε antibody (2C-11) for 72 hours in DMEM + 5% FCS at 37° C. Cells were harvested and incubated with Fc block (2.4G2 antibody). Dead cells were labeled using near-IR LIVE/DEAD Fixable Dead Cell Stain Kit (Invitrogen). Cells were stained with PerCP-Cy5.5-anti-CD4 (BioLegend); stained for intracellular Foxp3 and analyzed by flow cytometry as outlined above.

### Statistical analysis

All data are expressed as mean and SEM unless otherwise noted in the legend. Statistical significance was determined using GraphPad Prism 5.0 as indicated in the legends. Differences were considered significant at *p* < 0.05.

## Results

### CD8^+^ induced lethal lung injury is dependent on IL-2 levels during CD8 T cell differentiation

**Figure 1:**
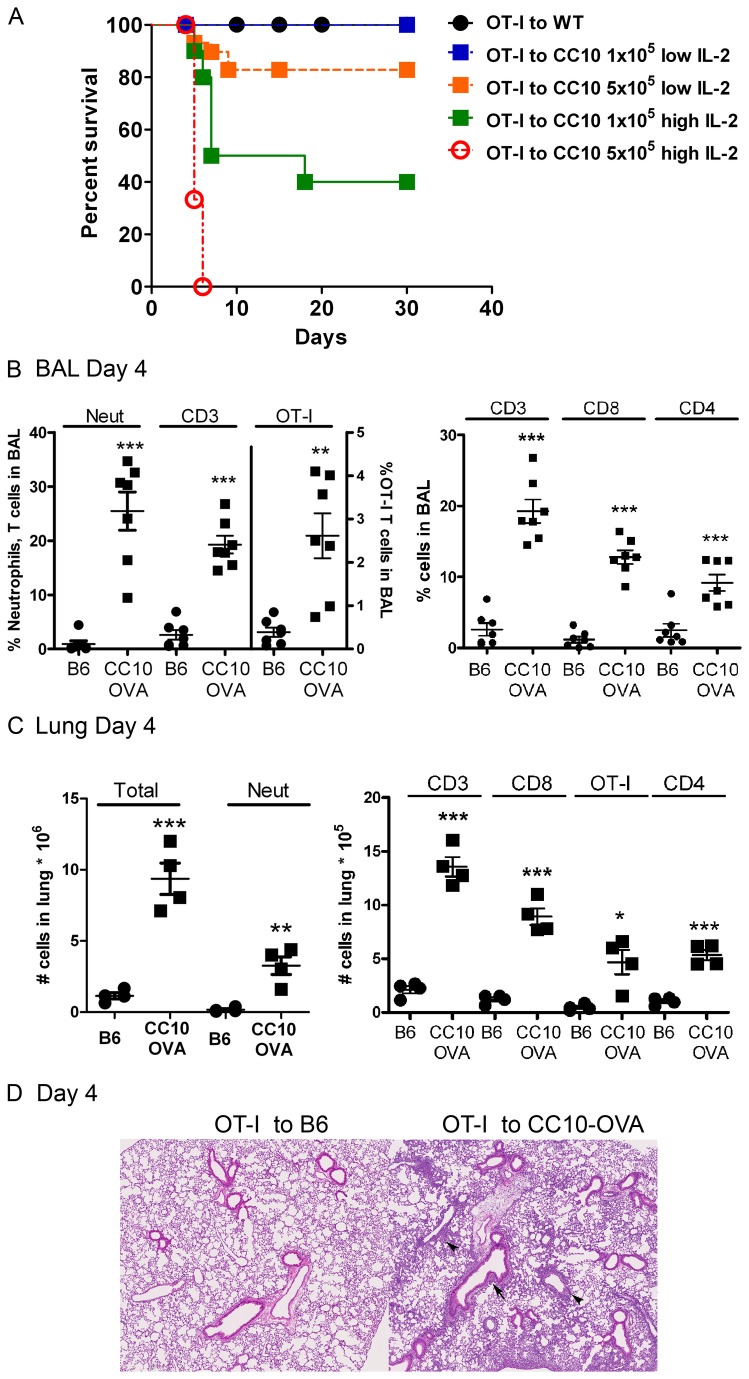
Acute lung inflammation induced by antigen-specific CD8^+^ T cells cultured with low dose IL-2 resolves. CC10-OVA. Thy1.2 transgenic mice or controls (B6.Thy1.2) were injected i.v. with the indicated numbers of activated OT-I. Thy1.1 T cells expanded with either high dose IL-2 (25U/ml) or low dose IL-2 (12.5U/ml). (A) Survival was dependent on the number of OT-I transferred and the dose of IL-2 given during culture. (B) The percentage of neutrophils (gated by size and Gr1^Hi^), CD3^+^Thy1.2^+^ and OT-I. Thy1.1^+^ T cells were measured in the BAL. (C) The numbers of cells in the lungs were measured on Day 4 after transfer of OT-I T cells. (D) Lung histology 4 days after transfer demonstrates peribronchial and perivascular mononuclear infiltrates in CC10-OVA mice compared to B6 mice; 7 µm sections were stained with H and E, magnification, 10X. Unpaired t tests were used to determine significance between controls (B6) and CC10-OVA mice, *p<0.05, **p<0.01, ***p<0.0001.

As previously shown, CC10-OVA mice adoptively transferred with 5x10^5^ activated OT-I CTL die within 6 days due to severe lung injury, while transfer of a lower amount of OT-I cells (1x10^5^) decreases mortality (Figure 1) [32,36]. As the mice receiving 1x10^5^ OT-I cells still had significant mortality with only 40% surviving, we wanted to develop a model where lung injury was induced but the majority of mice survived. This would allow us to dissect the mechanisms promoting and controlling CTL-mediated injury in the lung. CD8 T cell effector function and cytotoxicity have previously been shown to be highly dependent on the amount of IL-2 available during differentiation [37–40]. We found that decreasing the amount of IL-2 in the OT-I cultures from 25U/ml (high IL-2) by half to 12.5U/ml (low IL-2) at the time of culture expansion on Day 3, significantly altered the lethality of the transferred OT-I. Transfer of 5x10^5^ OT-I cells cultured with low IL-2 resulted in significantly decreased mortality compared to the 5x10^5^ OT-I cultured under high IL-2 conditions and transfer of 1x10^5^ OT-I cultured with low dose IL-2 did not induce death in any of the mice. The lethality of CTL in this model was not only dependent on the number of cells transferred but the level of IL-2 given to the CTL at the time of culture expansion.

### Acute lung inflammation mediated by activated antigen-specific CD8^+^ T cells recruits bystander T cells

While the CC10-OVA mice injected with 1x10^5^ low dose IL-2 OT-I cells survived, an acute inflammatory response was still evident in the lungs. There was an increase in the frequency of T cells, neutrophils, and transferred OT-I T cells in the BAL and lungs of CC10-OVA mice compared to B6 controls (Figure 1B, 1C). The immune response in the lungs of the CC10-OVA mice was also marked by an increase in endogenous (Thy1.2) CD8^+^ and CD4^+^ T cells (Figure 1B). The lungs of the CC10-OVA mice compared to controls had moderate perivascular and peribronchial inflammatory cell infiltration on day 4 (Figure 1D). By Day 30 the inflammation had completely resolved (data not shown). These data suggested neutrophils and lymphocytes recruited to the lung were playing a role in the injury induced by CD8^+^ T cells.

### CD4^+^ T cells are required for the resolution of acute lung inflammation

**Figure 2:**
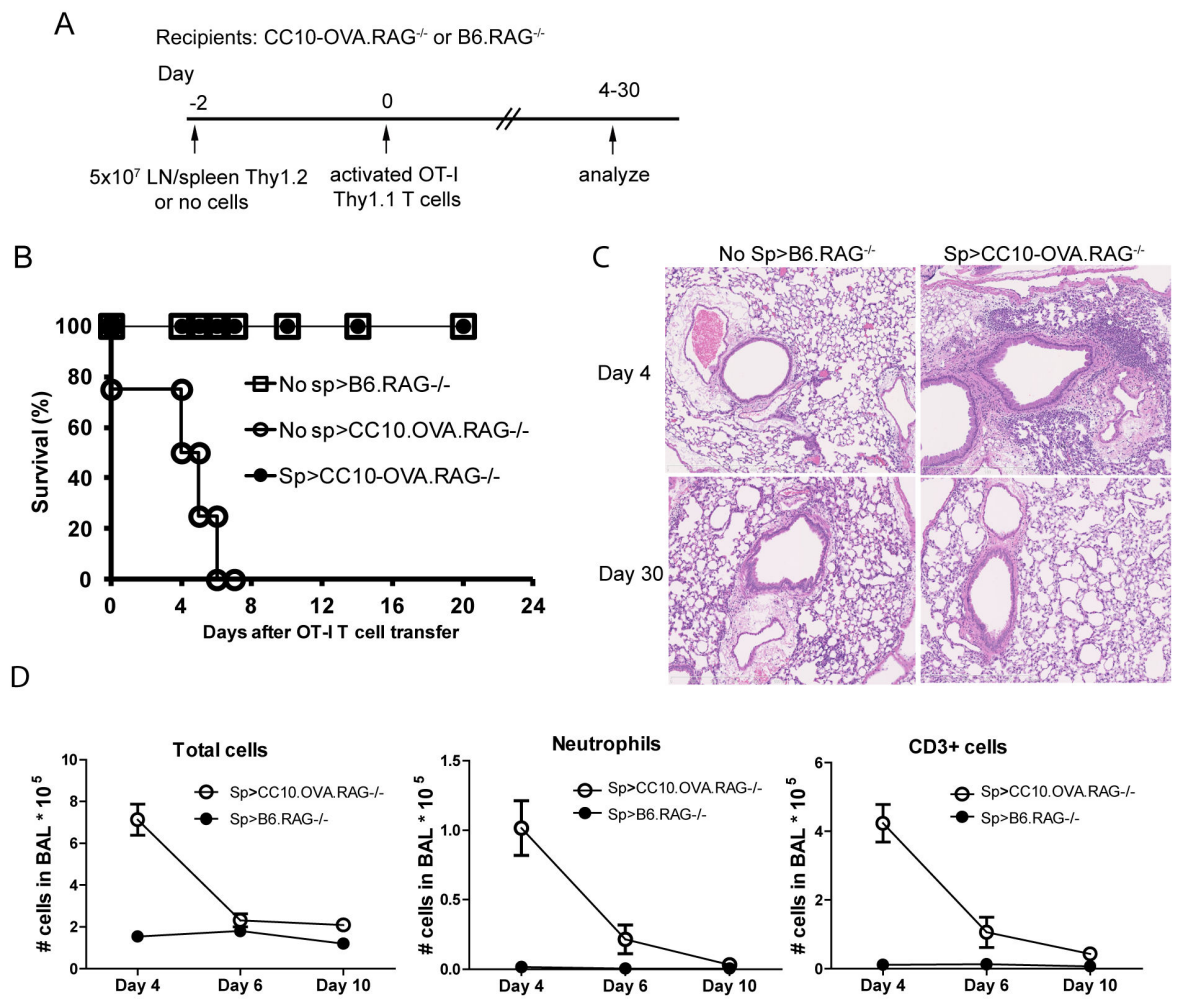
Bystander lymphocytes are required to resolve lung injury mediated by OT-I. CC10-OVA.RAG^-/-^ or B6.RAG^-/-^ mice were given 1x10^5^ OT-I activated in vitro with low dose IL-2 on Day 0. On Day -2, some animals received 5x10^7^ lymph node (LN) and spleen cells from B6 wild-type mice. Mice were sacrificed and lungs and BAL were analyzed at different time-points as indicated. (A) Experimental plan is outlined. (B) CC10-OVA.RAG^-/-^ mice that received only OT-I T cells died within 7 days (No sp > CC10-OVA.RAG^-/-^). CC10-OVA.RAG^-/-^ mice that received LN/spleen cells 2 days prior to transfer of OT-I survived (Sp > CC10-OVA.RAG^-/-^). Control mice were not affected (No Sp > B6.RAG^-/-^). (C) Lung tissue was harvested from CC10-OVA.RAG^-/-^ given LN/spleen cells prior to OT-I transfer and B6.RAG^-/-^ mice on days 4 and 30 after transfer of OT-I T cells. Sections (7 µm) were stained with H and E. Original magnification: 10X. (D) BAL from CC10-OVA.RAG^-/-^ mice given LN/spleen cells prior to OT-I transfer and controls was harvested and analyzed on days 4, 6, and 10 after adoptive transfer of 1x10^5^ OT-I T cells. Results are the mean ± SEM, CC10-OVA. RAG^-/-^ mice (n=3), B6.RAG^-/-^ (n=3) at each time-point.

To test the hypothesis that lymphocytes recruited to the lung were contributing to the inflammatory response in the CC10-OVA mice, we bred the CC10-OVA mice to B6.RAG^-/-^ to generate B6.CC10-OVA.RAG^-/-^ (Thy1.2) and B6.RAG^-/-^ (littermate control, Thy1.2) mice. The requirement for bystander lymphocytes to induce CD8-mediated lung injury was investigated by transferring 1x10^5^ activated OT-I. Thy1.1 T cells (low IL-2) into CC10-OVA.RAG^-/-^ mice or controls as outlined in Figure 2. All of the CC10-OVA.RAG^-/-^ (No Sp > CC10.OVA.RAG^-/-^) mice died after OT-I transfer but none of the control mice (No Sp > B6.RAG^-/-^) (Figure 2B). We expected that the inflammation may be less in the absence of other lymphocytes but instead the bystander lymphocytes recruited to the lung were protective. One explanation for this finding was that uncontrolled homeostatic proliferation contributed to death. Previous work by Surh and colleagues suggested that 50x10^6^ whole splenocytes diminish homeostatic proliferation in RAG^-/-^ mice [41]. To prevent homeostatic proliferation and determine the role of bystander lymphocytes, 50x10^6^ splenocytes from B6 (Thy1.2) mice were transferred two days prior to transfer of activated OT-I. Thy1.1 T cells, see timeline (Figure 2A). All CC10-OVA.Rag^-/-^ mice given bulk B6 lymph node/spleen cells (Sp > CC10.OVA.RAG^-/-^) survived for 30 days (Figure 2B). Perivascular and peribronchial inflammatory cell infiltrates were found in the lungs similar to that seen in CC10-OVA mice (Figure 2C). The BAL also had increased numbers of total cells, CD3^+^ T cells and neutrophils compared to controls (Figure 2D). The cell counts were highest at Day 4 and decreased to the level of controls by Day 10. These data were consistent with the day 10 lung pathology where few infiltrates remain. Complete resolution was found by Day 30 (Figure 2C). Lungs or airways from control B6.Rag^-/-^ mice on day 4 and day 30 did not have any significant cellular infiltration (Figure 2C, 2D).

**Figure 3:**
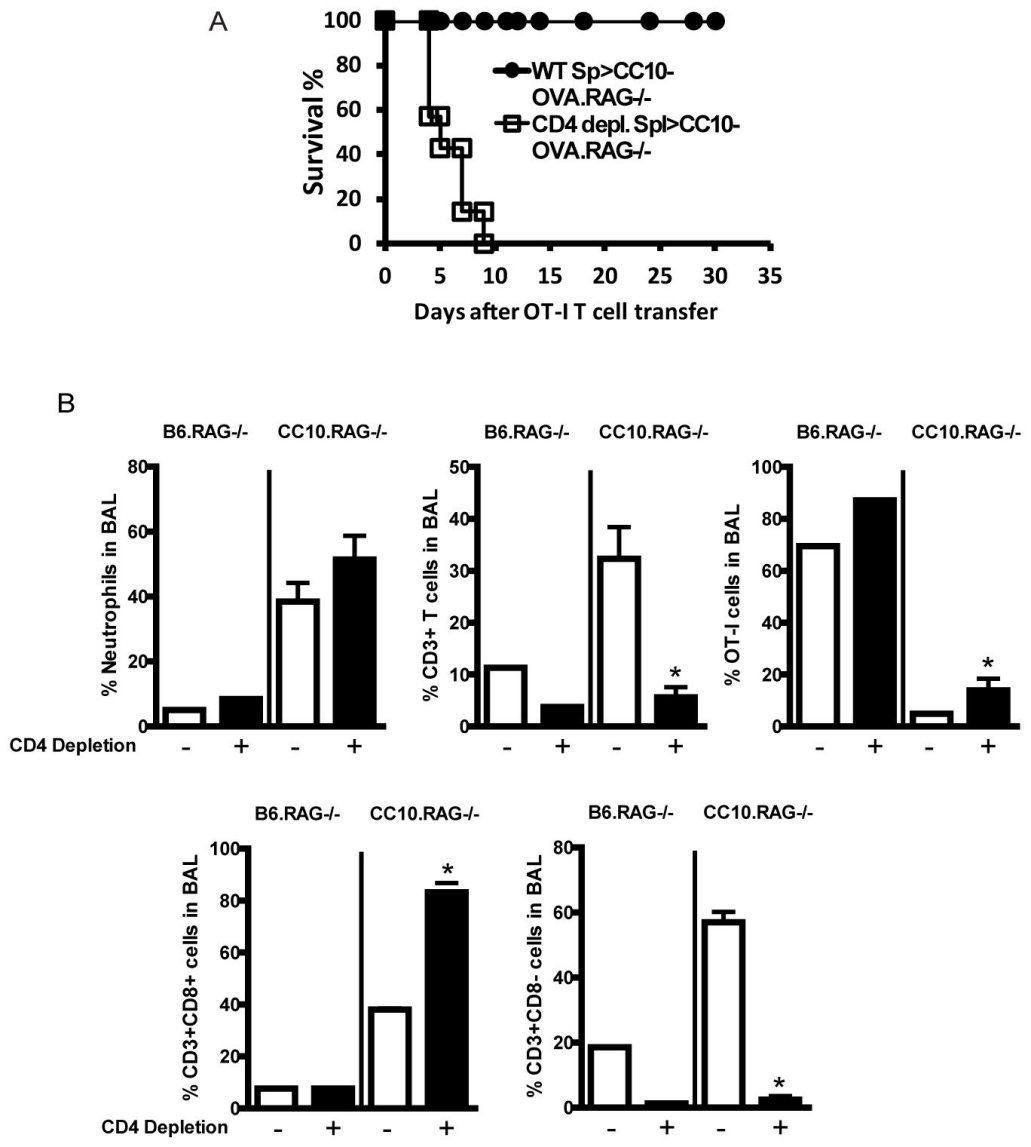
CD4^+^ T cells are required to prevent lethal lung injury. CC10-OVA. RAG^-/-^ mice were injected with LN/spleen cells or CD4 depleted LN/spleen Thy1.2 cells 2 days prior to i.v. injection of activated 1x10^5^ OT-I. Thy1.1 T cells. (A) Survival was dependent on CD4^+^ T cells. (B) Day 4 after OT-I transfer, frequency of BAL cells was measured in B6.RAG^-/-^ compared to CC10.OVA.RAG^-/-^ with or without CD4 depletion of LN/spleen cells.

Given the increase in CD4^+^ T cells in the B6.CC10-OVA mice, we investigated whether CD4^+^ T cells were playing a role in the inflammatory response. CC10-OVA.RAG^-/-^ mice received either bulk lymphocytes (combined lymph node/spleen) or CD4-depleted lymphocytes two days prior to i.v. injection of OT-I. Thy1.1 T cells. All CC10-OVA.RAG^-/-^ mice given CD4-depleted splenocytes died within 8 days, while the majority of the mice given bulk splenocytes survived (Figure 3A). These data suggest that bystander CD4^+^ T cells are necessary for resolution of acute lung inflammation in our model.

### Neutrophils are not required for CD8^+^ T cells to induce lethal lung injury

**Figure 4:**
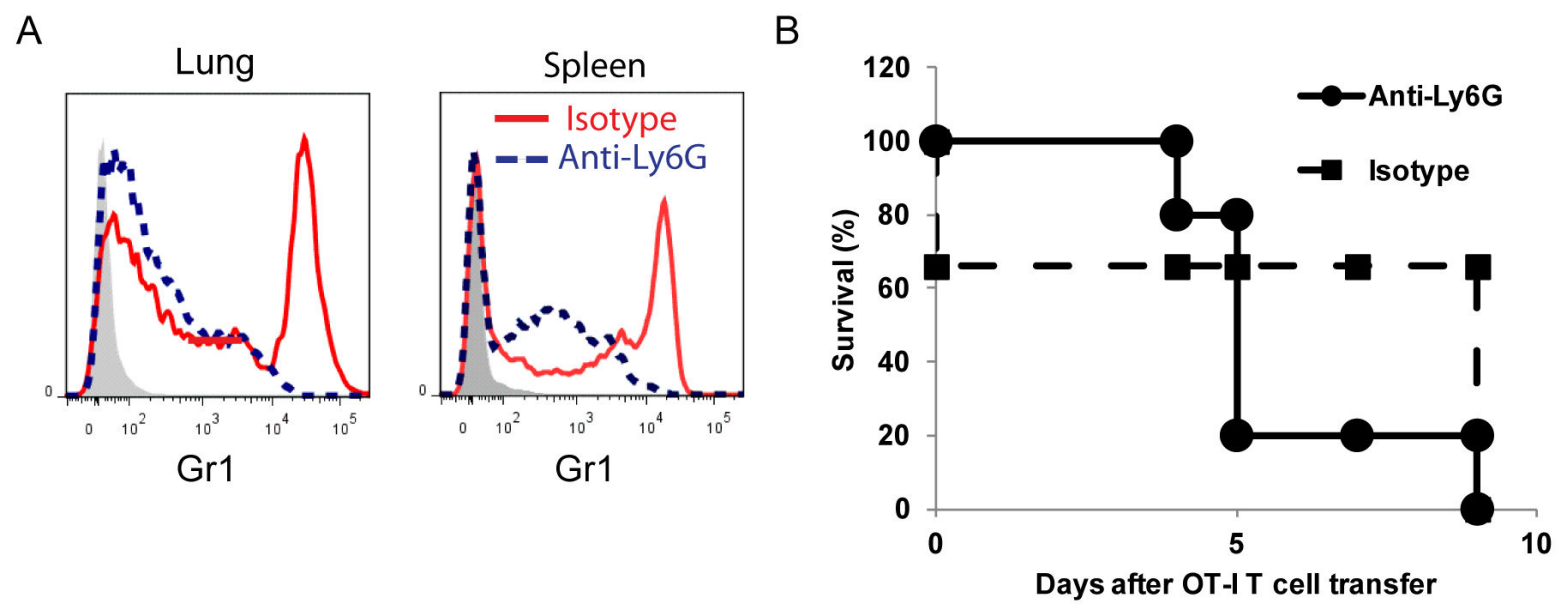
Neutrophils are not required for lethal lung injury. CC10-OVA.RAG^-/-^ mice were injected with anti-Ly6G antibody or isotype control antibody i.p. two days prior to transfer of OT-I T cells and on days 3 and 5 after OT-I transfer. (A) Anti-Ly6G antibody (dotted line) specifically depleted neutrophils (Gr1^Hi^) cells in both the lung and spleen as demonstrated by the absence of the Gr1^Hi^ peak by histogram compared to isotype control treated mice (solid line). Gray histogram is negative control for Gr1 staining. (B) No difference in mortality between anti-Ly6G treated animals and isotype control treated animals was found.

To determine the mechanism by which the CD4^+^ T cells may be regulating the inflammation in the lung we evaluated the BAL on Day 4 at the peak of inflammation and before mortality increased. In the absence of CD4^+^ T cells there was a significant increase in the percentage of OT-I T cells and a trend toward increased neutrophils (Figure 3B). As expected in the absence of CD4^+^ T cells there were more Thy1.2 ^+^ CD8^+^ T cells than Thy1.2 ^+^ CD8^-^ T cells (Figure 3B). While the percentage of neutrophils was not significantly increased it was still possible that neutrophils were contributing to the lethal lung injury by persisting in the CC10-OVA.RAG^-/-^ mice given OT-I T cells alone or CD4-depleted lymphocytes. CD4^+^ Treg have previously been found to regulate neutrophil apoptosis in a mouse model of LPS induced acute lung injury [42]. To test this possibility we depleted neutrophils using anti-Ly6G (1A8) antibody, known to specifically deplete neutrophils and not monocytes [43–45]. In Figure 4A, we found that injection of anti-Ly6G was sufficient to deplete neutrophils, defined as Gr1^Hi^ and also CD11b^+^Ly6G^+^ (data not shown), in the lungs and spleen compared to the isotype control antibody (2A3). However, neutrophil depletion in the CC10-OVA.RAG^-/-^ mice given OT-I T cells did not protect the mice from death (Figure 4B). Neutrophils were not required for CD8-mediated lethal lung injury in our model.

### CD4^+^CD25^+^Foxp3^+^ T cells in the lungs are ICOS^Hi^

**Figure 5:**
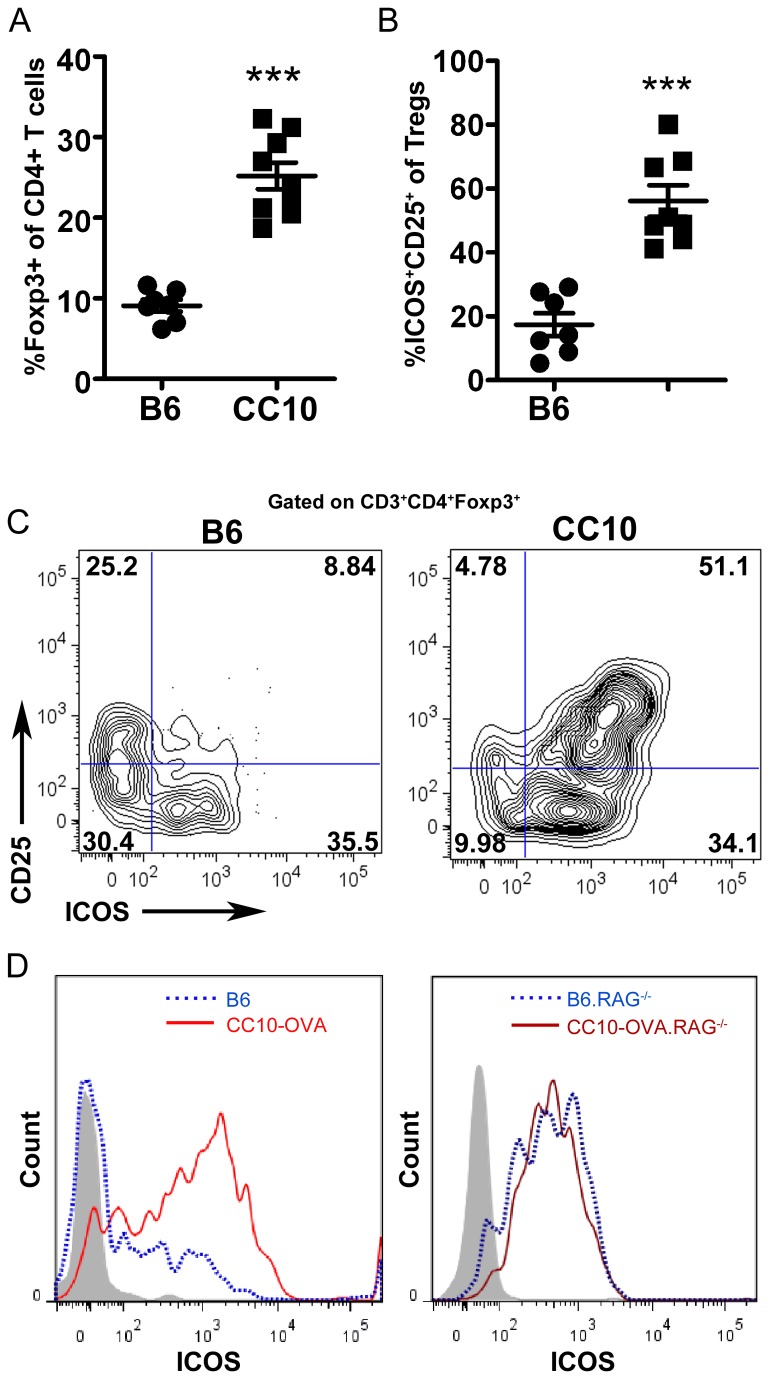
ICOS^+^ Tregs are increased in lungs of mice with CD8-mediated acute lung injury. Lung cells were harvested and analyzed by flow cytometry on day 4 after adoptive transfer of 1x10^5^ OT-I T cells (low dose IL-2). (A) Frequency of CD4^+^ Foxp3^+^ Tregs in the lungs. (B) Frequency of ICOS ^+^ CD25^+^ CD4^+^Tregs in the lung; data are representative of three independent experiments. (C) Representative FACS plot from lungs of control or CC10.OVA mice. (D) Left panel is ICOS expression on CD4^+^Foxp3^+^ T cells from lungs of B6 (dotted line) and B6.CC10-OVA (solid line). Right panel is ICOS expression on CD4^+^Foxp3^+^ Tregs from lungs of B6.RAG^-/-^ (dotted line) and B6.CC10-OVA.RAG^-/-^ mice (solid line).

In B6.CC10-OVA mice we had found a significant increase in CD4^+^ T cells in the lungs and BAL after transfer of activated OT-I T cells (Figure 1A, 1B). Previous work in this model has found a role for CD4^+^Foxp3^+^ Tregs in regulating the inflammatory response in the lung [36]. We also confirmed that the percentage and number of CD4^+^Foxp3^+^ Treg cells was significantly higher in B6.CC10-OVA mice compared to control B6 mice (Figure 5A and data not shown). Since our previous work has shown a role for ICOS in CD4^+^ Th2 effector function and migration to the lungs and others have found that ICOS expression regulates Treg function, we compared ICOS expression to CD25 expression on Treg in the lung [29,30,46,47]. We found that the frequency and absolute numbers of ICOS ^Hi^CD25^Hi^ Tregs were significantly higher in the lungs of CC10-OVA mice than wild-type mice (Figure 5 B, C and data not shown). Further, the majority of Treg from CC10-OVA mice (~80%) expressed high levels of ICOS (Figure 5C, D). In contrast, control B6 mice had on average ~40% ICOS^Hi^ Treg and Treg were either ICOS^Hi^ or CD25^Hi^ (Figure 5C,D). These data suggest that in response to inflammation in the lung Treg are activated and as a consequence upregulate ICOS along with CD25. Interestingly, when we evaluated ICOS expression on CD4^+^Foxp3^+^ Treg after transfer into B6.RAG^-/-^ controls or CC10-OVA.RAG^-/-^ mice given activated OT-I (schema in Figure 2), no difference in ICOS expression was found between the two groups. Thus, ICOS expression may be induced on Tregs in response to homeostatic proliferation irrespective of inflammatory signals.

### ICOS^-/-^ lymphocytes do not protect from CD8-mediated lung injury

**Figure 6:**
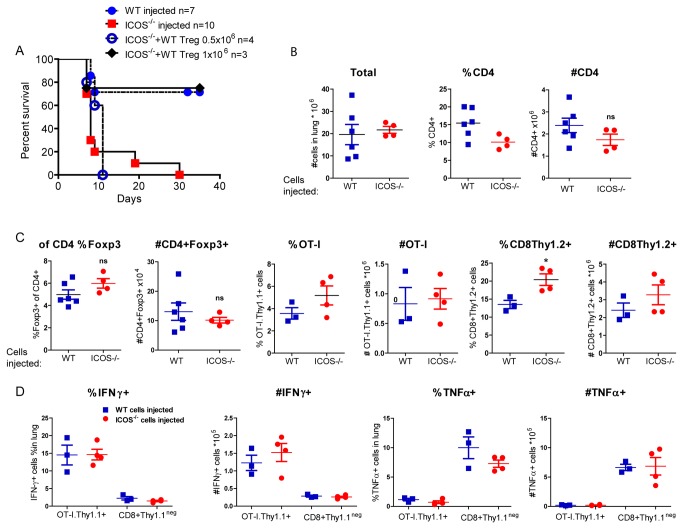
ICOS^-/-^ lymphocytes do not protect from CD8-mediated lung injury in mice. CC10-OVA.RAG^-/-^ mice were injected with wild-type LN/spleen cells, ICOS deficient LN/spleen cells, or ICOS deficient LN/spleen cells co-transferred with 0.5x10^6^ or 1x10^6^ ICOS^+/+^ Tregs 2 days prior to i.v. injection of activated 1x10^5^ OT-I T cells. (A) Survival (B) Total lung cells, percentage and absolute numbers of CD4^+^, CD4^+^Foxp3^+^ T cells. (C) Percentage and absolute number of OT-I. Thy1.1^+^ cells and CD8^+^ Thy1.2^+^ T cells. (D) Percentage and absolute number of OT.I. Thy1.1^+^ T cells and CD8^+^Thy1.2^+^ T cells expressing IFNγ or TNFα in the lungs at Day 4 were measured.

Since ICOS was highly expressed on Tregs during the inflammatory response in the lungs, we tested the ability of T cells from ICOS^-/-^ mice to inhibit lethal lung injury. Bulk splenocytes from ICOS^-/-^ mice or wild-type B6 mice were transferred into CC10-OVA.RAG^-/-^ mice prior to the transfer of activated OT-I T cells as outlined in Figure 2. The majority of mice that received ICOS^-/-^ splenocytes died within 8 days and all ICOS^-/-^ recipients were dead by 30 days (Figure 6A). On day 4 after OT-I transfer, the lungs from mice given WT or ICOS^-/-^ cells prior to OT-I transfer had similar numbers of total cells and there were no significant differences in the percentages of neutrophils (Figure 6B and data not shown). In addition, there were no differences in the frequency or number of CD4^+^, CD4^+^Foxp3^+^, or OT-I. Thy1.1^+^ T cells between the two groups. There was a significant difference in the frequency of non-OT-I.CD8 ^+^ Thy1.2^+^ T cells in the absence of ICOS but the absolute numbers of these cells was not different. There were also no differences in the frequency of IFNγ or TNFα producing OT-I T cells or CD8^+^Thy1.2 cells. To determine if the defect in the mice receiving ICOS^-/-^ splenocytes was specifically due to a defect in Tregs, we transferred 5x10^5^ or 1x10^6^ CD4^+^CD25^+^ Tregs from wild-type mice along with ICOS^-/-^ splenocytes. Transfer of 1x10^6^ wild-type Tregs but not 5x10^5^ wild-type Tregs was sufficient to prevent death and resolve the acute lung inflammation with the majority of mice alive at 30 days (Figure 6A). These data suggested that ICOS^-/-^ Tregs were defective in regulating CD8-mediated lung immunopathology. 

### ICOS^-/-^ Tregs have defects in IL-10 production and proliferation

**Figure 7:**
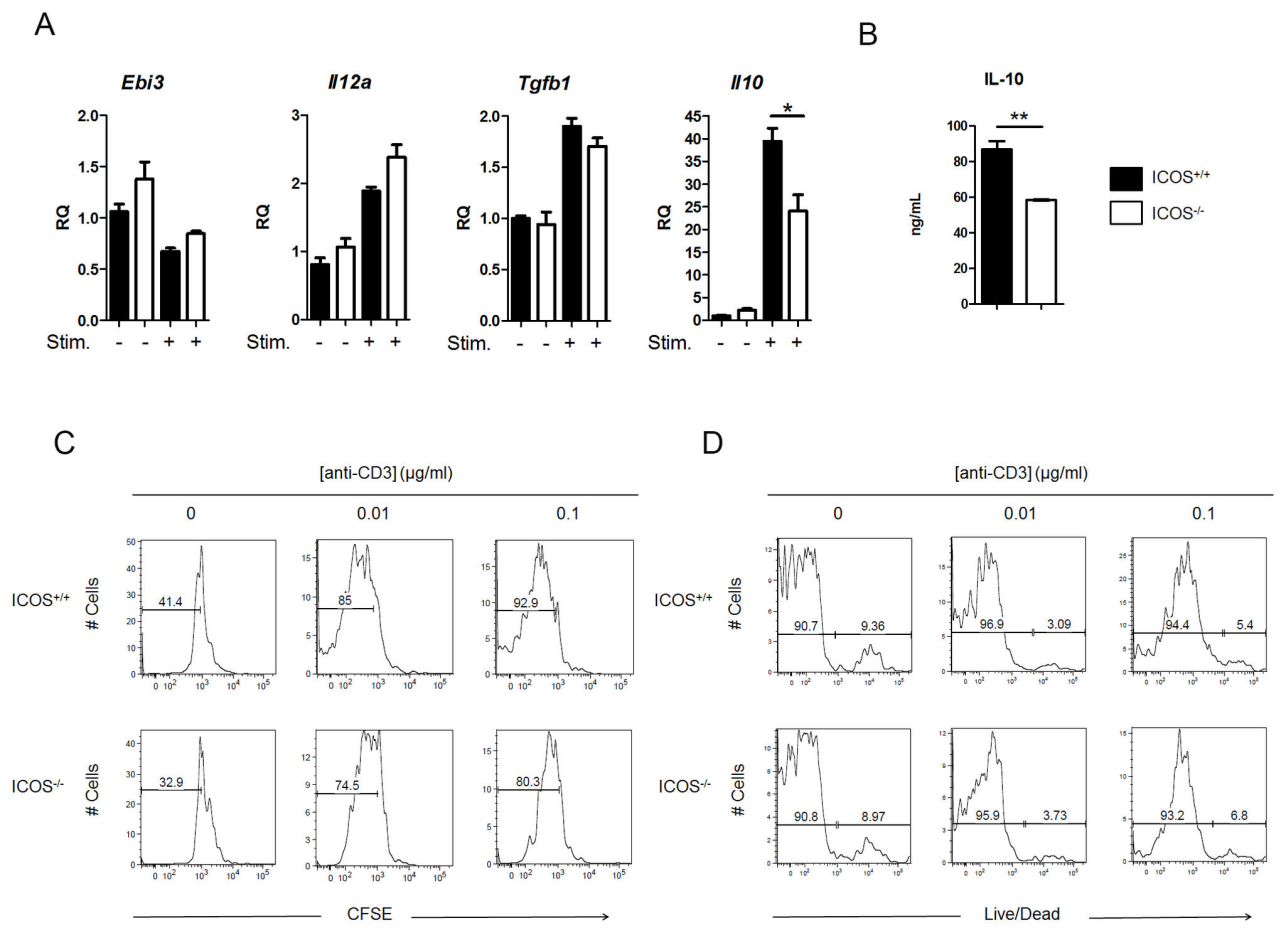
ICOS^-/-^ Tregs have defects in IL-10 production and proliferation. CD4^+^CD25^+^ Treg were isolated from C57Bl/6 ICOS^+/+^ and ICOS^-/-^ mice and stimulated for 72 hours in vitro with anti-CD3 and IL-2 (A and B). A) Regulatory cytokine expression was measured by qRT-PCR. (B) IL-10 in the culture supernatants was measured by ELISA. C and D: single-cell suspensions of spleen and lymph node cells from C57Bl/6 ICOS^+/+^ and ICOS^-/-^ mice were CFSE labeled, cultured for 72 hours in the presence of anti-CD3, and analyzed by flow cytometry. C. Percentage of CD4^+^Foxp3^+^ T cells that were CFSE^Lo^ was measured. D. Survival of CD4^+^Foxp3^+^ T cells from ICOS^+/+^ and ICOS^-/-^ mice was determined using a fixable live/dead dye (*p < 0.05, **p<0.01 – unpaired Student’s t test).

To further investigate the effect of ICOS deficiency on Tregs, we compared expression of the regulatory cytokines IL-10, IL-35, and TGF-β1 in unstimulated and stimulated ICOS^+/+^ Treg to ICOS^-/-^ Treg. Treg have been found to utilize IL-10 and TGF-β1 to suppress dendritic cell and T cell function and high ICOS expression has been associated with IL-10 producing Treg [29,48]. IL-35, a novel heterodimeric cytokine comprised of the IL-12α (p35) and EBV-induced gene product (EBI-3) subunits, has also been associated with high ICOS expressing Treg in a mouse model of airway hyperresponsiveness [49]. When stimulated in vitro, ICOS^-/-^ CD4^+^CD25^+^ T cells expressed significantly less *Il10* than ICOS^+/+^ CD4^+^CD25^+^ T cells (Figure 7A). However, no significant differences were observed in expression of the subunits of IL-35, *Ebi3* or *Il12a*, or *Tgfb1*, suggesting ICOS^-/-^ Treg have no defect in IL-35 or TGF-β1 expression (Figure 7A). In addition, ICOS^-/-^ CD4^+^CD25^+^ T cells secreted significantly less IL-10 than ICOS^+/+^ Treg (Figure 7B). No differences were observed in ex vivo cytokine expression levels between ICOS^+/+^ and ICOS^-/-^ CD4^+^CD25^+^ T cells (Figure 7A).

While ICOS has been previously linked to IL-10 expression, ICOS expression has also been associated with differences in Treg proliferation and survival. Chen, et al. identified two sub-populations of murine Treg: an ICOS^Hi^ hyperproliferative subset and an ICOS^Lo^ death-prone subset [50]. These results suggested ICOS^-/-^ Tregs may have defects in proliferation and survival. We compared the proliferative capacity and susceptibility to death in vitro of CD4^+^Foxp3^+^ Tregs from ICOS^+/+^ to ICOS^-/-^ mice. We found a slight decrease in proliferative capacity with ICOS^-/-^ Tregs (Figure 7C). This defect was specific to ICOS^-/-^ CD4^+^Foxp3^+^ Treg and was not observed in ICOS^-/-^ CD4^+^Foxp3^-^ conventional T cells (data not shown). There was no defect in survival of CD4^+^Foxp3^+^ Treg from ICOS^-/-^ mice compared to wild-type (Figure 7D). Taken together, these results suggest that ICOS expression is important for the proliferation potential, but not survival, of Treg.

### No defect of ICOS^-/-^ CD4^+^CD25^+^ Treg in vivo when directly compared with wild-type Treg

**Figure 8:**
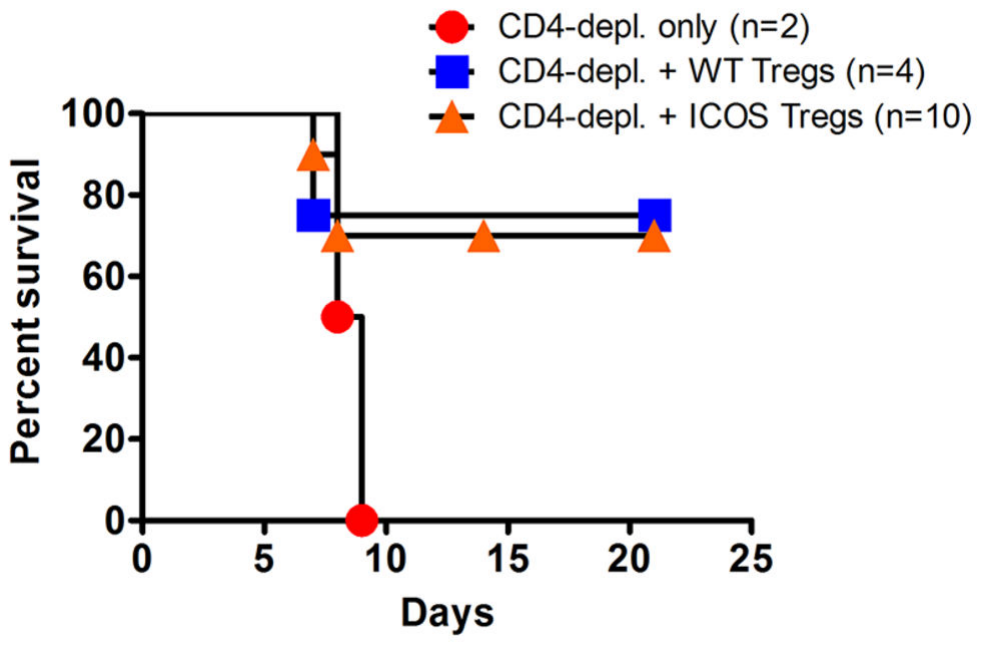
ICOS^-/-^ Tregs protect from lethal lung injury. CC10-OVA.RAG^-/-^ mice were injected with CD4-depleted wild-type LN/spleen cells in combination with 1x10^6^ ICOS^+/+^CD4^+^CD25^+^ or ICOS^-/-^CD4^+^CD25^+^ Tregs 2 days prior to i.v. injection of activated 1x10^5^ OT-I T cells. Survival was compared.

While our data suggested that ICOS^-/-^ CD4^+^CD25^+^ Tregs have a defect in vivo and in vitro, one possibility for our in vivo findings was that ICOS^-/-^ mice have less Tregs than wild-type mice [28,51]. In our experiments with transferring bulk lymph node and splenocytes, we estimated that 5x10^5^ Treg were being transferred. However, in Figure 6, we found that ICOS^+/+^ 5x10^5^ CD4^+^CD25^+^ Treg were not sufficient to protect the mice receiving ICOS^-/-^ lymphocytes while 1x10^6^ Treg did protect. To directly compare equal numbers of ICOS^+/+^ and ICOS^-/-^ Treg in vivo in our model in the absence of other CD4^+^ Treg, 1x10^6^ CD4^+^CD25^+^ Treg from ICOS^+/+^ or ICOS^-/-^ mice were transferred along with CD4-depleted lymphocytes from wild-type mice into CC10-OVA.RAG^-/-^ mice 2 days prior to the transfer of activated OT-I. These mice were compared to mice that received only CD4-depleted splenocytes. As found in Figure 3, CD4-depleted splenocytes were not protective from lethal lung injury (Figure 8). Contrary to expectations, we did not find a difference in survival between mice that received ICOS^+/+^ or ICOS^-/-^ Treg. There were no differences in the time to maximum weight loss or the overall weight loss in the animals that survived in either group (data not shown). These data suggest that ICOS^-/-^ CD4^+^CD25^+^ Treg can protect from lethal lung injury when sufficient numbers are transferred.

## Discussion

In a model of lung rejection, we have found that mice given a low dose of activated antigen-specific CD8^+^ T cells have reproducible lung inflammation that requires bystander lymphocytes to express ICOS for resolution of lung inflammation. In the absence of exogenous lymphocytes, CC10-OVA.RAG^-/-^ mice succumbed to lethal lung rejection from activated OT-I T cells. CD4^+^ T cells were required for resolution and ICOS^-/-^ lymphocytes were not sufficient to protect the CC10.OVA.RAG^-/-^ mice from the lethal lung injury. Wild-type Tregs were sufficient to rescue this defect. Tregs from ICOS^-/-^ mice had defects in proliferation and IL-10 expression and protein in vitro. However, adoptive transfer of ICOS^-/-^ Tregs directly compared to the same number of ICOS^+/+^ Tregs found no differences in the ability to protect from lethal lung injury. Our data suggest ICOS regulates the development of Tregs, but ICOS is not required for Treg effector function in our model.

Our work extends previous work in this model demonstrating that the balance between effector T cells (Teff) and Treg regulate protection from lethal lung injury in B6.CC10-OVA mice [36]. In addition, to differences in the numbers of Teff promoting lethal lung inflammation we found that modulating IL-2 levels during CTL differentiation affected the lethality of CTL in inducing lung injury. These data are consistent with studies demonstrating the role of IL-2 in augmenting CD8 effector function and cytotoxicity through increasing the transcription factor, eomesodermin (Eomes), and repressing BCL6 [39]. Thus the quantity of CTL and the quality of their effector functions determine the outcome of injury. As previously reported, the quantity of Tregs transferred affected their ability to prevent lethal lung inflammation [36]. Therapies that expand Tregs while downregulating the activation and trafficking of Teff may prevent acute rejection.

Our findings support the role of ICOS in Treg development, but do not confirm a role for ICOS in natural Treg effector function. This is despite the fact that Tregs from B6.CC10-OVA mice with lung rejection expressed high levels of ICOS and ICOS^+/+^ Treg, when present in sufficient numbers, were able to prevent death in mice that received ICOS-deficient bystander lymphocytes. These data suggested that ICOS would be essential for Tregs to suppress lethal lung injury and were consistent with previous studies using ICOS^-/-^ mice [48,51]. In contrast to expectations, we found that mice that received equal numbers of ICOS^-/-^ Tregs compared to wild-type Tregs did not succumb to lethal lung injury. Our data suggest that the deficiency found with bystander ICOS^-/-^ lymphocytes may be related to a defect in Treg development and the numbers of Treg, rather than a defect in their effector function in vivo in this model. This would be consistent with findings in a previous study that ICOS^-/-^ mice could develop functional CD4^+^ Treg in a model of tolerance [52].

Alternatively, bystander ICOS^-/-^ lymphocytes may be contributing to the lung injury. There was a significant increase in the frequency of CD8^+^Thy1.2^+^ T cells in mice that received ICOS^-/-^ lymphocytes compared to mice that received wild-type lymphocytes, but there was no difference in the absolute number of these cells. Further, no differences were detected in the frequency of ICOS^-/-^CD8^+^Thy1.2^+^ T cells producing TNFα, the major cytokine produced by these cells, or IFNγ, which only a few of these cells produced. There were very low numbers of dual producers. In addition, at the peak of inflammation, no differences were found in the expansion of the OT-I T cells in mice that received ICOS^-/-^ lymphocytes compared to mice that received wild-type lymphocytes. Thus, we did not find conclusive evidence of augmented responses in vivo from the bystander ICOS-deficient T cells or the transferred CTL to suggest ICOS^-/-^ lymphocytes were contributing to the lung injury. Another possibility is that Tregs function via other lymphocytes which are defective in ICOS^-/-^ mice, which has been suggested by others [52]. However, we found that ICOS^+/+^ Tregs were effective in controlling inflammation in the presence of ICOS^-/-^ lymphocytes which would make this possibility less likely. More studies are needed to determine the contribution of ICOS^-/-^ lymphocytes to the lung injury in our model.

The differences between ICOS^Hi/+^ and ICOS^Lo/-^ Tregs have been of interest in the literature and several groups have shown in both humans and mice differences in cytokine production and proliferation between these two subsets of Tregs [29,30,48,53]. Human ICOS^+^ Tregs produced higher levels of IL-10 and TGFβ than Tregs with low ICOS expression ex vivo and similar results were found in a mouse model of contact hypersensitivity [29,30]. Recent work has also associated IL-35 production with high ICOS expression on wild-type Treg [49]. In addition, ICOS expression was associated with a higher proliferative and survival capacity of Treg in vitro [50]. We investigated whether ICOS deficiency would affect cytokine expression, proliferation and survival of Treg in vitro. Consistent with previous studies we found that ICOS^-/-^ Treg had decreased IL-10 mRNA and protein after in vitro stimulation compared to ICOS^+/+^ Treg [51,53,54]. However, we did not find evidence of a likely defect in IL-35. In addition, we found a minimal defect in proliferation in vitro for ICOS^-/-^ Treg compared to ICOS^+/+^ Treg and no defects in survival. These data suggest that high ICOS expression may distinguish a subset of Tregs in wild-type mice, but ICOS is not necessary for all of the characteristics of these Tregs. Instead, ICOS expression may be a marker of full activation of Tregs or previous activation. This is consistent with the known induction of ICOS upon T cell activation. Interestingly, under homeostatic conditions in B6.RAG^-/-^ mice, all Treg were ICOS^Hi^ despite a portion being ICOS^Lo^ prior to transfer. Although we cannot exclude that ICOS^Hi^ Treg preferentially expand under these conditions, we think it is possible that ICOS can be upregulated on all Treg upon activation and proliferation. Inflammatory conditions and homeostatic proliferation appear to provide the signals necessary for Treg to upregulate ICOS expression or for ICOS^+^ Treg to expand.

In conclusion, we have found that tissue injury in the lung induces recruitment of bystander lymphocytes and an influx of activated Tregs, which express high levels of ICOS. With the right balance of Tregs to CTL lung homeostasis can be restored without the induction of fibrosis or any evidence of chronic inflammation. Strategies that tip the balance in favor of Tregs may be more protective of lung allografts than global immunosuppression. Furthermore, ICOS regulates the ability of bystander lymphocytes to resolve CTL mediated lung injury and may be an important target for modulating lung rejection. The mechanism by which ICOS affects bystander lymphocyte function requires more investigation and may be due to defects in the numbers of Tregs in ICOS^-/-^ mice. While ICOS is an important regulator of Treg development, ICOS is not necessarily required for Treg effector function in the lung.
